# The Predictive Value of Tumor Mutation Burden on Efficacy of Immune Checkpoint Inhibitors in Cancers: A Systematic Review and Meta-Analysis

**DOI:** 10.3389/fonc.2019.01161

**Published:** 2019-11-05

**Authors:** Yongfeng Wu, Jinming Xu, Chengli Du, Yihua Wu, Dajing Xia, Wang Lv, Jian Hu

**Affiliations:** ^1^Department of Thoracic Surgery, The First Affiliated Hospital, School of Medicine, Zhejiang University, Hangzhou, China; ^2^Department of Toxicology, School of Public Health, Women's Hospital, School of Medicine, Zhejiang University, Hangzhou, China

**Keywords:** tumor mutation burden, tumor mutational burden, immune checkpoint inhibitors, objective response rate, progression-free survival, overall survival, meta-analysis

## Abstract

**Background:** Despite an increasing understanding about tumor mutation burden (TMB) in cancer immunity and cancer immunotherapy, the comprehensive cognition between TMB and efficiency of immune checkpoint inhibitors (ICIs) is still lacking. A systematic review and meta-analysis was conducted to evaluate the predictive value of TMB on efficacy of ICIs.

**Methods:** Systematic literature search was conducted on PubMed, EMBASE, Web of Science and Cochrane Library up to June 16, 2019. Pooled odds ratio (OR) of objective response rate (ORR), hazard ratio (HR) of progression-free survival (PFS) and overall survival (OS) were estimated by inverse variance weighted fixed-effects model (*I*^2^ ≤ 50%) or DerSimonian-Laird random-effects model (*I*^2^ > 50%). In addition, heterogeneity analysis, sensitivity analysis, publication bias and subgroup analysis were conducted. Moreover, fractional polynomial regression was conducted to investigate the dose-response relationship between TMB cutoffs and efficacy of ICIs. Furthermore, we assessed ORR by TMB and programmed cell death ligand 1 (PD-L1) expression after layering each other in studies which the two could be both acquired.

**Results:** Three thousand six hundred fifty-seven records were retrieved through database searching, and 29 studies with 4,431 patients were finally included in the meta-analysis. TMB high group had significantly improved ORR (pooled OR 3.31, 95% CI 2.61, 4.19, *P* < 0.001), PFS (pooled HR 0.59, 95% CI 0.49, 0.71, *P* < 0.001) and OS (pooled HR 0.68, 95% CI 0.53, 0.89, *P* = 0.004). Sensitivity analyses illustrated the results were stable, and publication bias was identified in ORR. Subgroup analyses showed the predictive value of TMB was significant in non-small-cell lung cancer (except for the OS) and melanoma. In addition, heterogeneity was substantial in targeted next generation sequencing group but tiny in whole exome sequencing group. Furthermore, TMB and PD-L1 expression were capable to predict improved ORR of ICIs after stratification of each other, with tiny heterogeneity.

**Conclusions:** High tumor mutation burden predicted improved efficacy of immune checkpoint inhibitors in cancers, and targeted next generation sequencing for estimating tumor mutation burden in clinic should be standardized to eliminate heterogeneity in the future. Moreover, tumor mutation burden and programmed cell death ligand 1 expression were independent factors on predicting efficacy of immune checkpoint inhibitors.

## Introduction

Immune checkpoint inhibitors (ICIs) have been identified to improve response and survival in diverse solid tumors and hematologic malignancies, including melanoma, non-small-cell lung cancer (NSCLC), urothelial carcinoma, renal-cell carcinoma and Hodgkin's lymphoma (1–6). However, the efficacy seems unsatisfactory in unselected patients (1, 3, 7), suggesting eligible biomarkers are required to identify subgroups appropriate for cancer immunotherapy. At present, scientists have recognized several candidate biomarkers, such as programmed cell death ligand 1 (PD-L1) expression, tumor-infiltrating lymphocytes (TILs), transcriptomic and epigenetic signatures, oncogenic driver mutations and mismatch repair deficiency (dMMR)/microsatellite instability (MSI) (8–10). Among them, tumor mutation burden (TMB), which is defined as the number of mutations (generally non-synonymous somatic mutations) in cancer cells, is likely to be a promising biomarker. It has been reported that patients with high TMB have better response and survival to ICIs than patients with low TMB in melanoma, NSCLC and urothelial carcinoma (11–16). Recently, Samstein et al. have utilized a large cohort of 1,662 patients to validate that high TMB is capable of forecasting preferable overall survival in multiple cancer types (17). Moreover, Singal et al. exploited real-world data from an electronic health records database, further verifying the predictive capability of TMB in NSCLC (18). Furthermore, TMB is widely recognized as a biomarker independent of PD-L1 expression (18–20).

There are quite a lot of evidences supporting the function of TMB. It is associated with neoantigen burden (13, 21), which can activate T lymphocytes to proliferate and kill cancer cells (8). In addition, tumors with dMMR generate a mass of somatic mutations and exhibit MSI which present high TMB (22–24), and dMMR/MSI is connected with response to ICIs (22, 23, 25).

Despite a number of studies uncovering powerful forecasting capability of TMB on efficacy of ICIs, however, negative results are also reported, especially in long-term survival (26–29). Several reasons may explain the heterogeneity of these results. Firstly, since TMB is not significant in all caners (30), it may have predictive value in particular cancer types. Besides, due to diverse cut-off values adopted in different studies (14, 26, 31, 32), the optimum TMB threshold in a wide range of cancers or a typical cancer type is still a mystery. In addition, owing to huge cost and complexity of whole exome sequencing (WES), targeted next generation sequencing (NGS) has been widely adopted to evaluate TMB of cancer cells. However, significant heterogeneity could exist due to quite a lot of variables in different gene panels (33).

Although there are two meta-analyses reporting the predictive value of TMB, the number of studies and patients included is small, and subgroup analyses are insufficient to explain heterogeneity of the results (34, 35). Hence, we did a more comprehensive systematic review and meta-analysis to evaluate the influence of tumor mutation burden on efficacy of immune checkpoint inhibitors in cancers, and conduct overall subgroup analyses to identify potential source of heterogeneity.

## Methods

### Data Sources, Search Strategy, and Selection Criteria

The PRISMA statement was followed in the systematic review and meta-analysis (36). Systematic literature search was conducted on PubMed, EMBASE, Web of Science and Cochrane Library up to June 16, 2019. Two investigators (Wu and Xu) searched the databases independently. The search term was as follows: (PD-1 OR PD-L1 OR CTLA-4 OR Ipilimumab OR Tremelimumab OR Nivolumab OR Pembrolizumab OR Lambrolizumab OR Atezolizumab OR Avelumab OR Durvalumab OR “immune checkpoint inhibitor” OR “immune checkpoint inhibitors” OR “ICI” OR “ICIs” OR “immune checkpoint blocker” OR “immune checkpoint blockers” OR “ICB” OR “ICBs”) AND (mutation burden OR mutational burden OR mutation load OR mutational load OR TMB OR TML). When duplicate reports were identified, the one with larger sample size and more detailed information was selected. We also reviewed references in articles finally included to identify studies potentially missed.

To be eligible, studies had to meet the following inclusion criteria: (1) cohort studies or clinical trials assessed inhibitors of PD-1/PD-L1, CTLA-4, or their combination, in patients with cancer, and the efficiency of therapy was evaluated by TMB which had cut-off value; (2) odds ratio (OR) of objective response rate/overall response rate (ORR), or hazard ratio (HR) of progression-free survival (PFS) or overall survival (OS), and their 95% confidence intervals (95% CI) were given in the article, or sufficient data was available to calculate them; (3) the number of patients accessible for evaluation was no <20; (4) studies were published in English. Reviews, notes, letters, editorials, comments, meeting abstracts, and case reports were excluded on account of insufficient information.

Two investigators (Wu and Xu) independently reviewed the retrieved studies to identify potential applicable articles, and any disagreements about specific articles were discussed and determined with consensus of all investigators.

### Data Extraction and Quality Assessment

Two investigators (Wu and Xu) independently extracted data from studies included, and any inconsistencies were conferred and resolved with consensus of all investigators. The following information was extracted from each study: title, first author, year of publication, type of cancer, study design, data source, sample size evaluable for TMB, area of patients, class of immune checkpoint inhibitors, line of therapy, median age, gender, TMB sequencing method, TMB cut-off value, outcomes (ORR, PFS, OS) and their value. When duplicate publications were identified, the most comprehensive one was included.

The Newcastle-Ottawa Scale (NOS) was adopted to assess the quality of studies included (37). The total score ranged from 0 to 9, as 8–9 points indicated high quality of a study, five to seven points indicated medium quality, and studies with points lower than five showed poor quality.

### Data Synthesis and Statistical Analysis

The primary endpoint of the meta-analysis was the comparison on efficiency of ICIs between TMB high group and TMB low group, which was measured in terms of OR of ORR, and HR of PFS and OS. Heterogeneity among individual studies was evaluated by the Q test; *I*^2^ > 50% and/or *P* ≤ 0.10 indicated significant heterogeneity (38). Pooled OR or HR with Z test was calculated by DerSimonian-Laird random-effects model when significant heterogeneity was identified, otherwise inverse variance weighted fixed-effects model was adopted. In addition, funnel plots were constructed, and Begg's test and Egger's test were performed to evaluate publication bias (*P* ≤ 0.10 was considered to be visible publication bias). Besides, sensitivity analysis was used to test the stability of the results in the meta-analysis. To further explore variation of effect of TMB on immunotherapy efficiency, subgroup analyses stratified by cancer type, area of patients, TMB sequencing method, class of immune checkpoint inhibitors, and line of therapy were conducted. Moreover, to investigate the dose-response relationship between TMB cutoffs and efficacy of ICIs, fractional polynomial regression (two degree) was conducted on studies of no <50 patients. To note, total mutation burden detected by WES was converted to mutations per megabase using a linear transformation (39). Furthermore, we evaluated ORR by TMB and PD-L1 expression after layering each other in studies which the two could be both acquired. Stata version 11.0 (Stata Corporation, College Station, TX) was used for analyses mentioned above.

In particular, there were several articles providing original data or graphs without reporting OR or HR. For original response data, STATA 11.0 was used to estimate OR. For original survival data, SPSS 20.0 was used to calculate HR through a Cox proportional hazards regression model. For Kaplan–Meier curves, Engauge Digitizer was used to extract survival data from graphs, then HR was estimated by adopting the method reported by Tierney et al. (40).

## Results

### Study Characteristics and Data Quality

**Figure 1:**
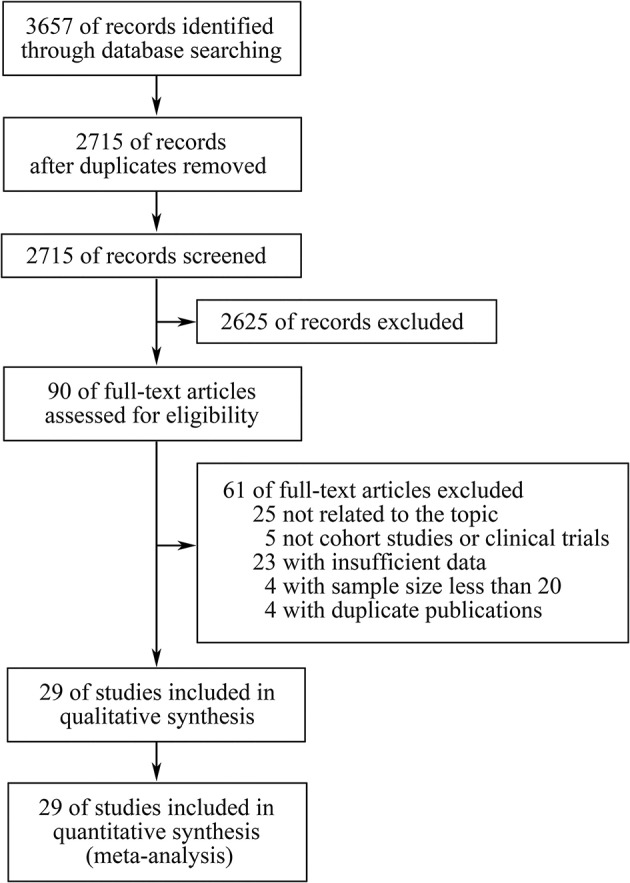
The PRISMA flow diagram.

Three thousand six hundred fifty-seven records were retrieved through database searching, from which 90 studies potentially relevant to our topic were identified through screening of titles and abstracts. Subsequently, after full-text screening and qualitative synthesis, 29 studies with 4,431 patients were finally included in the meta-analysis (11–14, 17, 19, 20, 26–29, 31, 32, 41–56), including 26 cohort studies and three clinical trials (Figure 1; Table 1; Supplementary File 1: Table S1). In particular, four duplicate reports (57–60), two studies assessing TMB as a continuous variable (61, 62), and four studies with sample size <20 (63–66) were identified and excluded. There were 11 studies for patients with NSCLC, eight for melanoma, three for gastroesophageal cancer, two for small cell lung cancer (SCLC), two for diverse cancers, one for colorectal cancer, one for melanoma or urologic cancers, and one for three independent cohorts which were pan-tumor, HNSCC and melanoma, respectively. In these studies, 20 articles researched patients in Western countries, six articles investigated patients in Asia, and three articles studied patients in multiple areas. Different classes of ICIs were studied, including 18 studies for anti-PD-(L)1 monotherapy, four for anti-CTLA-4 monotherapy, two for anti-PD-1 in combination with anti-CTLA-4, and four studies comprised anti-PD-(L)1 monotherapy or in combination with anti-CTLA-4. In particular, there was another one study including two independent cohorts with dissimilar classes of ICIs: one was anti-PD-1 monotherapy, the other was anti-PD-1 in combination with anti-CTLA-4. In terms of line of therapy, two studies were done in first-line settings, and 18 studies were done in multiple lines, whereas the rest nine studies didn't mention the line. WES was adopted to detect TMB in 13 studies, and targeted NGS was used in the remaining studies. For the former, TMB was determined by the total number of mutations, and for the latter, TMB was defined as the number of mutations per megabase except for one article which derived the predicted total mutation load (PTML). To note, there were two studies using blood tumor mutation burden (bTMB), one study adopting circulating tumor deoxyribonucleic acid (ctDNA) TMB, and three studies dividing TMB into three segments in which the high TMB group and the low TMB group were included while the medium TMB group was excluded. The results of Newcastle-Ottawa Scale were listed in Supplementary File 1: Table S2. There were seven studies having a high quality, and the remaining studies had a medium quality, which ensured relative high quality of the studies included and enhanced reliability of the meta-analysis. To note, the randomized trial reported by Carbone et al. (53) was also assessed by NOS as patients simply treated with ICIs were included in the meta-analysis.

**Table 1:** Main characteristics of studies included in the meta-analysis.

**References**	**Cancer type**	**Sample size evaluable for TMB**	**Area**	**TMB sequencing method**	**Outcomes**
Wang et al. (38)	NSCLC	50	Asian	Targeted NGS	ORR, PFS
Van Allen et al. (39)	Melanoma	110	Western	WES	ORR^a^, PFS^a^, OS^a^
Teo et al. (14)	Urothelial carcinoma	60	Western	Targeted NGS	ORR, PFS, OS
Tang et al. (26)	Melanoma or urologic cancers	23	Asian	Targeted NGS	ORR, PFS^b^, OS^b^
Snyder et al. (11)	Melanoma	Discovery: 25; validation: 39	Multiple areas	WES	OS (discovery and validation cohorts)
Roszik et al. (40)	Melanoma	76	Western	Targeted NGS	OS
Roh et al. (41)	Melanoma	21	Western	WES	ORR^a^
Rizvi et al. (13)	NSCLC	34	Western	WES	ORR
Rizvi et al. (19)	NSCLC	240	Western	Targeted NGS	PFS
Ricciuti et al. (42)	SCLC	52	Western	Targeted NGS	ORR, PFS, OS
Riaz et al. (43)	NSCLC	68	Western	WES	ORR^a^, OS^a^
Ready et al. (44)	NSCLC	98	Western	Targeted NGS	ORR, PFS^b^
Morrison et al. (27)	Melanoma	160	Western	Targeted NGS	ORR^a^, OS^b^
Mishima et al. (28)	Gastric cancer	80	Asian	Targeted NGS	ORR, PFS
Huang et al. (29)	Esophageal carcinoma	23	Asian	WES	PFS
Huang et al. (46)	Gastric/ gastroesophageal junction cancer	20	Asian	WES	ORR
Hellmann et al. (20)	NSCLC	75	Western	WES	ORR, PFS
Hellmann et al. (47)	SCLC	Anti-PD-1: 133; anti-PD-1 plus anti-CTLA-4: 78	Western	WES	ORR, PFS^b^
Goodman et al. (48)	Diverse cancers	102	Western	Targeted NGS	ORR, PFS, OS
Cristescu et al. (50)	Pan-tumor, HNSCC, melanoma	Pan-tumor: 119; HNSCC: 107; melanoma: 89	Western	WES	ORR, PFS
Chae et al. (51)	NSCLC	82	Western	Targeted NGS	PFS, OS
Carbone et al. (52)	NSCLC	158	Multiple areas	WES	ORR, PFS^b^
Johnson et al. (12)	Melanoma	65	Western	Targeted NGS	ORR, PFS, OS
Hugo et al. (45)	Melanoma	38	Western	WES	ORR^a^, OS^a^
Samstein et al. (17)	Diverse cancers	1662	Western	Targeted NGS	OS^a^
Gandara et al. (49)	NSCLC	POPLAR: 105^a^; OAK: 324^a^	Multiple areas	Targeted NGS	ORR^a^, PFS^a^, OS^a^
Fang et al. (53)	NSCLC	73	Asian	WES	ORR, PFS
Schrock et al. (54)	Colorectal cancer	22	Western	Targeted NGS	ORR, PFS
Chae et al. (55)	NSCLC	20	Western	Targeted NGS	PFS, OS

a *The value was calculated from original data of the study*.

b *The value was calculated from Kaplan–Meier curves in the article*.

*NSCLC, non-small-cell lung cancer; SCLC, small cell lung cancer; HNSCC, head and neck squamous cell carcinoma; PD-1, programmed cell death 1; CTLA-4, cytotoxic T-lymphocyte-associated protein 4; TMB, tumor mutation burden; NGS, next generation sequencing; WES, whole exome sequencing; ORR, objective response rate/overall response rate; PFS, progression-free survival; OS, overall survival*.

### General Analysis of the Association Between TMB and Efficiency of Immune Checkpoint Inhibitors

**Figure 2:**
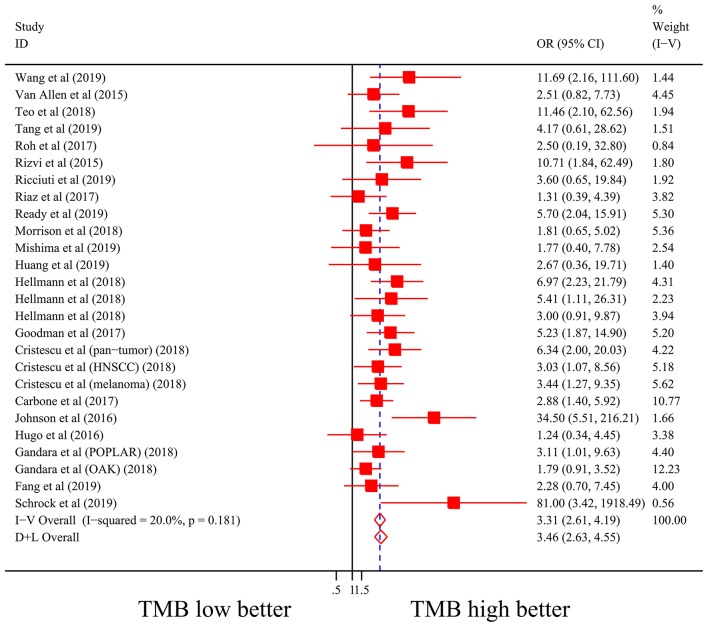
Forest plot of association between TMB and objective response rate of immune checkpoint inhibitors. OR, odds ratio; CI, confidence interval; TMB, tumor mutation burden.

**Figure 3:**
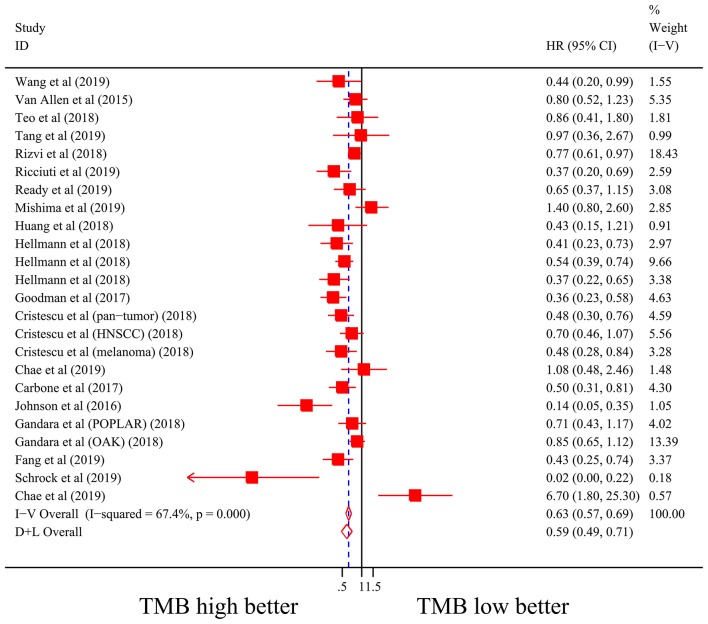
Forest plot of association between TMB and progression-free survival of immune checkpoint inhibitors. HR, hazard ratio; CI, confidence interval; TMB, tumor mutation burden.

**Figure 4:**
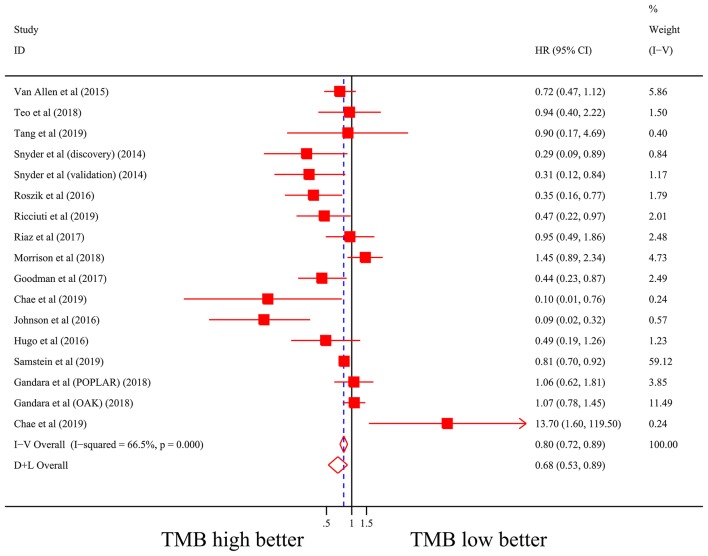
Forest plot of association between TMB and overall survival of immune checkpoint inhibitors. HR, hazard ratio; CI, confidence interval; TMB, tumor mutation burden.

Firstly, there were 22 studies (26 cohorts) including 2,013 patients evaluating the correlation between TMB and ORR of ICIs therapy. TMB high group had a significantly better ORR than TMB low group (pooled OR 3.31, 95% CI 2.61, 4.19, *P* < 0.001; Figure 2). The heterogeneity was insignificant (*I*^2^ = 20.0%, *P* = 0.181; Figure 2), and sensitivity analysis showed that the result was stable (Supplementary File 1: Figure S1a). Begg's test (*P* = 0.064; Supplementary File 1: Figure S1b) and Egger's test (*P* = 0.009; Supplementary File 1: Figure S1b) indicated publication bias was present. Since it seemed the publication bias was mostly caused by studies with small sample size, we re-analyzed the association after excluding eight studies with sample size no more than 50. TMB high group still showed an improved ORR (pooled OR 3.09, 95% CI 2.40, 3.97, *P* < 0.001), and the heterogeneity was tiny (*I*^2^ = 0%, *P* = 0.588). Interestingly, the publication bias disappeared (*P*_*Begg*_ = 0.405, *P*_*Egger*_ = 0.115). Secondly, the relationship between TMB and PFS of ICIs treatment was assessed in 20 studies (24 cohorts) containing 2,073 patients. PFS was evidently improved in TMB high group (pooled HR 0.59, 95% CI 0.49 0.71, *P* < 0.001; Figure 3), with a significant heterogeneity existing (*I*^2^ = 67.4%, *P* < 0.001; Figure 3). Sensitivity analysis showed a good stability of the pooled HR (Supplementary File 1: Figure S1c), and no publication bias was recognized (*P*_*Begg*_ = 0.673, *P*_*Egger*_ = 0.199; Supplementary File 1: Figure S1d). In addition, 15 studies (17 cohorts) including 2,936 patients were evaluated for the relevance between TMB and OS of ICIs. Patients with high TMB had a visibly superior OS than patients with low TMB (pooled HR 0.68, 95% CI 0.53, 0.89, *P* = 0.004; Figure 4). The result illustrated a significant heterogeneity (I^2^ = 66.5%, *P* < 0.001; Figure 4) and a good stability (Supplementary File 1: Figure S1e). Begg's test (*P* = 0.108; Supplementary File 1: Figure S1f) and Egger's test (*P* = 0.187; Supplementary File 1: Figure S1f) showed no publication bias.

### Subgroup Analyses and Fractional Polynomial Regression of the Association Between TMB and Efficiency of Immune Checkpoint Inhibitors

The results of subgroup analyses are shown in Table 2; Supplementary File 1: Table S3. Firstly, in terms of diverse cancer types, it was showed that in NSCLC, TMB high group had significantly better ORR (pooled OR 3.23, 95% CI 2.27, 4.59, *P* < 0.001) and PFS (pooled HR 0.65, 95% CI 0.50, 0.85, *P* = 0.001) than TMB low group, while no difference in OS (pooled HR 1.00, 95% CI 0.67, 1.50, *P* >.99) between the two groups was found. In melanoma, TMB high group had evidently improved ORR (pooled OR 2.55, 95% CI 1.60, 4.05, *P* < 0.001), PFS (pooled HR 0.46, 95% CI 0.23, 0.94, *P* = 0.033) and OS (pooled HR 0.55, 95% CI 0.37, 0.82, *P* = 0.004). In SCLC, superior ORR and PFS were found in TMB high group, while no result with statistical difference was discovered in urothelial carcinoma or gastroesophageal cancer. Secondly, in western countries, patients with high TMB had evidently better ORR, PFS and OS than patients with low TMB; and in Asia, TMB high group showed superior ORR but no better PFS than TMB low group. Besides, no matter whether TMB was measured by WES or targeted NGS, high TMB predicted improved ORR, PFS and OS of ICIs therapy. However, the former showed insignificant heterogeneity while the latter presented substantial heterogeneity. In addition, the efficiency was enhanced in TMB high group with anti-PD-(L)1 monotherapy, anti-CTLA-4 monotherapy or combined therapy, except for ORR and PFS in anti-CTLA-4 monotherapy as well as OS in combined therapy. Moreover, improvement of ORR and PFS was seen in TMB high group with first-line treatment of ICIs.

**Table 2:** Subgroup analyses of the association between TMB and efficiency of immune checkpoint inhibitors.

**Categories**	**ORR**		**PFS**		**OS**	
	**pooled OR (95% CI)**	***I*^**2**^ (%)**	**pooled HR (95% CI)**	***I*^**2**^ (%)**	**pooled HR (95% CI)**	***I*^**2**^ (%)**
**Cancer type**						
Non-small-cell Lung cancer	3.23 (2.27, 4.59)	18.1	0.65 (0.50, 0.85)	63.5	1.00 (0.67, 1.50)	59.4
Melanoma	2.55 (1.60, 4.05)	39.4	0.46 (0.23, 0.94)	72.6	0.55 (0.37, 0.82)	66.7
Small cell lung cancer	3.69 (1.60, 8.47)	0	0.47 (0.36, 0.60)	2.1	N/A	
Urothelial carcinoma	N/A		N/A		1.07 (0.79, 1.45)	0
Gastroesophageal cancer	2.05 (0.62, 6.75)	0	0.84 (0.27, 2.65)	73.2	N/A	
Others^a^	5.90 (3.27, 10.62)	2.8	0.53 (0.33, 0.85)	64.2	0.70 (0.57, 0.86)	0
**Area**						
Western	3.92 (2.90, 5.30)	29.1	0.55 (0.43, 0.71)	71.5	0.66 (0.47, 0.92)	69.8
Asian	2.96 (1.45, 6.06)	0	0.65 (0.37, 1.14)	63.1	N/A	
Others^a^	2.36 (1.50, 3.71)	0	0.74 (0.60, 0.92)	44.1	0.72 (0.43, 1.21)	59.5
**TMB sequencing method**						
WES	3.13 (2.28, 4.30)	0	0.53 (0.46, 0.62)	0	0.63 (0.46, 0.86)	32.1
Targeted NGS	3.55 (2.48, 5.07)	47.0	0.66 (0.48, 0.91)	75.7	0.73 (0.53, 1.02)	71.9
**Class of immune checkpoint inhibitors**						
Anti-PD-(L)1	3.26 (2.47, 4.29)	33.0	0.60 (0.47, 0.78)	70.6	0.71 (0.45, 1.14)	68.1
Anti-CTLA-4	2.51 (0.90, 7.02)	0	N/A		0.47 (0.36, 0.63)	38.8
Anti-PD-(L)1 plus anti-CTLA-4	5.04 (2.65, 9.59)	0	0.46 (0.33, 0.64)	9.2	N/A	
Others^a^	2.17 (0.90, 5.22)	0	0.66 (0.46, 0.96)	59.9	0.86 (0.53, 1.40)	73.3
**Line of therapy**						
1	3.61 (2.00, 6.51)	12.1	0.56 (0.39, 0.80)	0	N/A	
Others^a^	3.25 (2.51, 4.21)	23.4	0.59 (0.48, 0.73)	69.9	0.68 (0.53, 0.89)	66.5

a *Others included subgroups with only one report and articles containing multiple subgroups which could not be further subdivided*.

*ORR, objective response rate/overall response rate; PFS, progression-free survival; OS, overall survival; OR, odds ratio; HR, hazard ratio; CI, confidence interval; TMB, tumor mutation burden; WES, whole exome sequencing; NGS, next generation sequencing; PD-(L)1, programmed cell death 1/programmed cell death ligand 1; CTLA-4, cytotoxic T-lymphocyte-associated protein 4; N/A, not applicable*.

Moreover, to investigate the dose-response relationship between TMB cutoffs and efficacy of ICIs, fractional polynomial regression was conducted, and the results were shown in Supplementary File 1: Figure S2. Most studies had cutoffs between 5 and 10 muts/Mb. Within this range, the predictive OR of ORR and the predictive HR of PFS and their 95% CIs were meaningful and relatively stable. However, the predictive HR of OS and its 95% CI seemed meaningless within the entire range of cutoffs.

### TMB and PD-L1 Expression Were Independent Biomarkers to Predict Objective Response Rate of Immune Checkpoint Inhibitors

To explore whether TMB and PD-L1 expression were separate biomarkers to forecast efficiency of ICIs treatment, we identified 9 studies from articles included in the meta-analysis (13, 20, 26, 27, 31, 48, 51–53), which had sufficient data to calculate ORR in subgroups as follows: group 1, both high expression of TMB and PD-L1; group 2, both low expression of TMB and PD-L1; group 3, low expression of TMB and high expression of PD-L1; group 4, high expression of TMB and low expression of PD-L1. As shown in Table 3; Supplementary File 1: Table S4, patients with high TMB still had superior ORR than patients with low TMB after layering PD-L1 expression. Similarly, ORR could still be enhanced in PD-L1 expression high group after layering TMB level. All results dramatically showed tiny heterogeneity.

**Table 3:** TMB and PD-L1 expression were independent biomarkers to predict objective response rate of immune checkpoint inhibitors.

**Subgroup**	**TMB high vs. TMB low**		**PD-L1 high vs. PD-L1 low**	
	**Pooled OR (95%CI)**	***I*^**2**^ (%)**	**Pooled OR (95%CI)**	***I*^**2**^ (%)**
PD-L1 high group	3.13 (2.07, 4.75)	0	N/A	
PD-L1 low group	3.00 (1.72, 5.24)	0		
TMB high group	N/A		2.28 (1.37, 3.81)	6.1
TMB low group			2.97 (1.79, 4.92)	0

*TMB, tumor mutation burden; PD-L1, programmed cell death ligand 1; OR, odds ratio; CI, confidence interval; N/A, not applicable*.

## Discussion

The results of this study illustrated that high TMB was responsible for improved efficiency of ICIs therapy. It was significant in melanoma and NSCLC whose TMB level almost topped in diverse cancers (30, 67). However, the predictive value of TMB for long-term survival in NSCLC was still in doubt due to our negative result. Besides, high TMB could predict better ORR and PFS in SCLC, which required further research owing to insufficient number of studies and patients. Most studies were done in Western people, in which the strong association between high TMB and improved immunotherapy efficacy was identified, while more parallel research was required in Asian area. It seemed that high TMB could forecast enhanced efficiency of multiple classes of immune checkpoint inhibitors, especially combined therapy (anti-PD-(L)1 plus anti-CTLA-4). However, the result should be further confirmed due to most of the studies done in PD-(L)1 monotherapy.

Significant heterogeneity was detected in pooled PFS and OS, which could be partially explained by subgroup analyses of cancer type, class of immune checkpoint inhibitors and line of therapy. Interestingly, different TMB sequencing methods might clarify most of the heterogeneity, as it was concentrated in targeted NGS group. Though WES was used to detect TMB in initial studies which discovered that patients with high TMB responded better to ICIs (11, 13, 42), targeted NGS was subsequently widely applicated in research and clinic due to its comparative cheap cost and simplicity. To date, two targeted NGS panels have been approved by Food & Drug Administration (FDA) which are Memorial Sloan Kettering-Integrated Mutation Profiling of Actionable Cancer Targets (MSK-IMPACT) and FoundationOne CDx (F1CDx). However, our results suggested there was significant heterogeneity in dissimilar targeted NGS panels which might affect predictive accuracy and stability. Actually, panel-based TMB evaluation is affected by several experimental factors (e.g., tumor purity or sequencing depth) and the variant calling pipeline, which need to be standardized in different targeted NGS panels (33, 68). In addition, publication bias in pooled ORR should be considered. As the publication bias might be primarily caused by several studies with small sample size due to our results, further research with large sample volume and normative design was demanded.

Moreover, we identified that TMB and PD-L1 expression were capable to predict improved ORR of ICIs after stratification of each other, with dramatically tiny heterogeneity. As it was reported that TMB and PD-L1 expression could independently predict benefit to ICIs (19), our results further supported the view.

One of the most critical issues about TMB is the best threshold on predicting immunotherapy efficacy. Due to our results of fractional polynomial regression, most studies had cutoffs between 5 and 10 muts/Mb, which seemed to present a relative stable predictive value in multiple tumor analysis. However, the number of studies is far from enough to make a convincing conclusion, especially studies with cutoffs above 10 muts/Mb as well as studies reporting the long-term survival data. Actually, as TMB varies greatly in different tumors, there may not be a universal TMB cut-off value for all cancer types, especially cancers with high TMB level such as NSCLC and melanoma (17, 30). Encouragingly, a number of clinical trials are in progress in the context of TMB assessment in diverse cancers (33), which are expected to provide more high quality data to help us identify appropriate TMB cutoffs in certain cancer types.

Interestingly, there is another strategy to divide TMB into three layers, which are TMB high, medium and low groups (12, 49, 53), as the clinical benefit gap between TMB high group and TMB low group seems to be more significant. In addition, it has been reported that the three-tier TMB classification scheme can improve the accuracy of panel-based TMB measurement (69). Actually, there is a great deal of uncertainty on response to ICIs for patients whose TMB level is close to the cutoff. Therefore, the concept of medium TMB could clinically help doctors to comprehensively consider the treatment of such a population, which needs further research.

There are several strengths in our study. First of all, we adopted ORR, PFS and OS as our endpoints to evaluate both short-term and long-term benefits of ICIs therapy, which made it more comprehensive and convincing. Secondly, we did subgroup analyses from diverse aspects, and discovered most of the source of heterogeneity. In addition, sensitivity analyses showed a good stability of our results.

However, the current meta-analysis is restricted by several limitations. Firstly, sample size varied among the included studies, which resulted in large variance in sample volume between different subgroups, and quite a few studies with small sample quantity might be the chief source of publication bias in the meta-analysis. Moreover, a few important clinical characteristics, which has been reported to be responsible for efficiency of ICIs, such as age and sex (70, 71), were not corrected in several studies when calculating effect size.

There are four main conclusions drawn from our study. The first is that high TMB could predict improved efficiency of ICIs. It was significant in NSCLC and melanoma, but the predictive value on long-term survival of NSCLC requires further research. The second is that more studies with large sample size and standardized design are necessitated to further explore the prophetic worth of TMB in certain subgroups, especially in SCLC, Asian area and combined therapy (anti-PD-(L)1 plus anti-CTLA-4). Thirdly, targeted NGS for estimating TMB in clinic should be standardized to eliminate heterogeneity in the future. Moreover, we further validated that TMB and PD-L1 expression were independent factors on predicting response to ICIs. Therefore, the model combining TMB with PD-L1 expression may expand the group benefit from immune checkpoint inhibitors.

## Data Availability Statement

All datasets generated for this study are included in the article/Supplementary Material.

## Author Contributions

JH contributed to the conception and design of the work. YoW and JX contributed to conception, design, data analysis, and editing the manuscript. CD, YiW, DX, and WL contributed to data acquisition and critical revision of the manuscript. All authors read and approved the final manuscript.

### Conflict of Interest

The authors declare that the research was conducted in the absence of any commercial or financial relationships that could be construed as a potential conflict of interest.

## Abbreviations

TMB: tumor mutation burden
ICIs: immune checkpoint inhibitors
OR: odds ratio
ORR: objective response rate/overall response rate
HR: hazard ratio
PFS: progression-free survival
OS: overall survival
CI: confidence interval
PD-L1: programmed cell death ligand 1
NSCLC: non-small-cell lung cancer
TILs: tumor-infiltrating lymphocytes
dMMR: mismatch repair deficiency
MSI: microsatellite instability
WES: whole exome sequencing
NGS: next generation sequencing
NOS: Newcastle-Ottawa Scale
SCLC: small cell lung cancer
PTML: predicted total mutation load
bTMB: blood tumor mutation burden
ctDNA: circulating tumor deoxyribonucleic acid
FDA: Food & Drug Administration
MSK-IMPACT: Memorial Sloan Kettering-Integrated Mutation Profiling of Actionable Cancer Targets
F1CDx: FoundationOne CDx
HNSCC: head and neck squamous cell carcinoma
CTLA-4: cytotoxic T-lymphocyte-associated protein 4
N/A: not applicable
muts/Mb: mutations per megabase.
