# Supplementary material for: The Predictive Value of Tumor Mutation Burden on Efficacy of Immune Checkpoint Inhibitors in Cancers: A Systematic Review and Meta-Analysis
Source: Front Oncol. 2019 Nov 5;9:1161. doi: 10.3389/fonc.2019.01161 (PMC6848266; doi:10.3389/fonc.2019.01161)
Supplement: Supplementary file 1 [file Data_Sheet_1.PDF]

Table S1. Additional information of studies included in the meta-analysis

study	study design	class of immune checkpoint inhibitors	line	median age (years)	female (%)	TMB cut-off value
Wang et al (2019)[38]	cohort	anti-PD-(L)1	≥1	58	60	6 muts/Mb (bTMB)
Van Allen et al (2015)[39]	cohort	anti-CTLA-4	≥1	61.5[a]	29.1[a]	197[c]
Teo et al (2018)[14]	cohort	anti-PD-(L)1	≥1	67	11.7	ORR: 16.98 muts/Mb; PFS and OS: 7.35 muts/Mb
Tang et al (2019)[26]	phase 1 trial	anti-PD-1	≥2	51[b]	58.3[b]	6 muts/Mb
Snyder et al (2014)[11]	cohort	anti-CTLA-4	N/A	N/A	discovery: 35.9; validation: 44	100
Roszik et al (2016)[40]	cohort	anti-CTLA-4	N/A	59[a]	25[a]	100 (PTML)
Roh et al (2017)[41]	cohort	anti-CTLA-4	≥1	61[a]	33.3[a]	273[c]
Rizvi et al (2015)[13]	cohort	anti-PD-1	≥1	63	53	200
Rizvi et al (2018)[19]	cohort	anti-PD-(L)1 or plus anti-CTLA-4	≥1	66	51	7.4 muts/Mb
Ricciuti et al (2019)[42]	cohort	anti-PD-1 or plus anti-CTLA-4	≥2	65	51.9	9.68 muts/Mb
Riaz et al (2017)[43]	cohort	anti-PD-1	N/A	55	N/A	100
Ready et al (2019)[44]	phase 2 trial	anti-PD-1 plus anti-CTLA-4	1	65.5	44.9	10 muts/Mb
Morrison et al (2018)[27]	cohort	anti-PD-1 or plus anti-CTLA-4	N/A	61	33.1	7.1 muts/Mb
Mishima et al (2019)[28]	cohort	anti-PD-1	≥3	67	24	10 muts/Mb
Huang et al (2018)[29]	cohort	anti-PD-1	≥2	63[b]	6.7[b]	60

Huang et al (2019)[46]	cohort	anti-PD-1	≥2	60.5[b]	23.3[b]	70
Hellmann et al (2018)[20]	cohort	anti-PD-1 plus anti-CTLA-4	N/A	66	51	158
Hellmann et al (2018)[47]	cohort	anti-PD-1; anti-PD-1 plus anti-CTLA-4	≥2	anti-PD-1: 63; anti-PD-1 plus anti-CTLA-4: 65	anti-PD-1: 41; anti-PD-1 plus anti-CTLA-4: 33	high: 248; low: 143
Goodman et al (2017)[48]	cohort	anti-PD-(L)1	≥1	59	38	19 muts/Mb
Cristescu et al (2018)[50]	cohort	anti-PD-1	N/A	pan-tumor: 62; HNSCC: 61; melanoma: 60	pan-tumor: 56.3; HNSCC: 17.8; melanoma: 34.8	pan-tumor: 102.5; HNSCC: 86; melanoma: 191.5
Chae et al (2019)[51]	cohort	anti-PD-(L)1	≥1	64.5	59.8	15 muts/Mb
Carbone et al (2017)[52]	phase 3 trial	anti-PD-1	1	65	33.5	ORR: 243; PFS: high: 243; low: 100
Johnson et al (2016)[12]	cohort	anti-PD-(L)1	≥1	N/A	41.5	high: 23.1 muts/Mb; low: 3.3 muts/Mb
Hugo et al (2016)[45]	cohort	anti-PD-1	N/A	61.5[a]	28.9	489[c]
Samstein et al (2019)[17]	cohort	anti-PD-(L)1 or plus anti-CTLA-4	N/A	N/A	37.8[a]	5.9 muts/Mb[c]
Gandara et al (2018)[49]	cohort	anti-PD-L1	≥2	POPLAR: 61[a]; OAK: 63[a]	POPLAR: 31.4[a]; OAK: 37.3[a]	16 muts/Mb (bTMB)
Fang et al (2019)[53]	cohort	anti-PD-(L)1	N/A	55[a]	30.1[a]	157
Schrock et al (2019)[54]	cohort	anti-PD-(L)1	≥1	52	54.5	between 37 and 41 muts/Mb
Chae et al (2019)[55]	cohort	anti-PD-(L)1	≥1	66	60	7.2muts/Mb (ctDNA TMB)

[a]The value was calculated from original data in supplementary materials. [b]The value was taken from the whole cohort in the article. [c]Median TMB from original data in supplementary materials was recognized as cut-off value. Abbreviations: PD-(L)1: programmed cell death 1/programmed

cell death ligand 1. CTLA-4: cytotoxic T-lymphocyte-associated protein 4. HNSCC: head and neck squamous cell carcinoma. TMB: tumor mutation burden. muts/Mb: mutations per megabase. bTMB: blood tumor mutation burden. ORR: objective response rate/overall response rate. PFS: progression-free survival. OS: overall survival. PTML: predicted total mutation load. ctDNA: circulating tumor deoxyribonucleic acid. N/A: not applicable.

Table S2. Quality assessment of studies included in the meta-analysis using Newcastle-Ottawa Scale (NOS)

Study	Q1[a]	Q2	Q3	Q4	Q5	Q6	Q7	Q8	Total NOS score
Wang et al (2019)[38]	*	*	*	*	**	*	*	*	9
Van Allen et al (2015)[39]	*	*	*	*	*	*	*	*	8
Teo et al (2018)[14]	*	*	*	*		*	*	*	7
Tang et al (2019)[26]		*	*	*		*	*	*	6
Snyder et al (2014)[11]		*	*	*		*	*	*	6
Roszik et al (2016)[40]	*	*	*	*	*	*	*	*	8
Roh et al (2017)[41]		*	*	*	*	*	*	*	7
Rizvi et al (2015)[13]		*	*	*	*	*	*	*	7
Rizvi et al (2018)[19]	*	*	*	*		*	*	*	7
Ricciuti et al (2019)[42]	*	*	*	*	**	*	*	*	9
Riaz et al (2017)[43]	*	*	*	*		*	*	*	7
Ready et al (2019)[44]	*	*	*	*		*	*	*	7
Morrison et al (2018)[27]	*	*	*	*		*	*	*	7
Mishima et al (2019)[28]	*	*	*	*		*	*	*	7
Huang et al (2018)[29]		*	*	*		*	*	*	6
Huang et al (2019)[46]		*	*	*		*	*	*	6
Hellmann et al (2018)[20]	*	*	*	*	**	*	*	*	9
Hellmann et al (2018)[47]	*	*	*	*		*	*	*	7
Goodman et al (2017)[48]	*	*	*	*		*	*	*	7
Cristescu et al (2018)[50]	*	*	*	*		*	*	*	7

Chae et al (2019)[51]	*	*	*	*		*	*	*	7
Carbone et al (2017)[52]	*	*	*	*		*	*	*	7
Johnson et al (2016)[12]	*	*	*	*		*	*	*	7
Hugo et al (2016)[45]		*	*	*	*	*	*	*	7
Samstein et al (2019)[17]	*	*	*	*	*	*	*	*	8
Gandara et al (2018)[49]	*	*	*	*	*	*	*	*	8
Fang et al (2019)[53]	*	*	*	*		*	*	*	7
Schrock et al (2019)[54]		*	*	*		*	*	*	6
Chae et al (2019)[55]		*	*	*		*	*	*	6

[a]Q1: Representativeness of the exposed cohort; Q2: Selection of the non-exposed cohort; Q3: Ascertainment of exposure; Q4: Outcome of interest not present at start of study; Q5: Comparability of cohorts; Q6: Assessment of outcome; Q7: Follow-up long enough; Q8: Adequacy of follow up of cohorts.

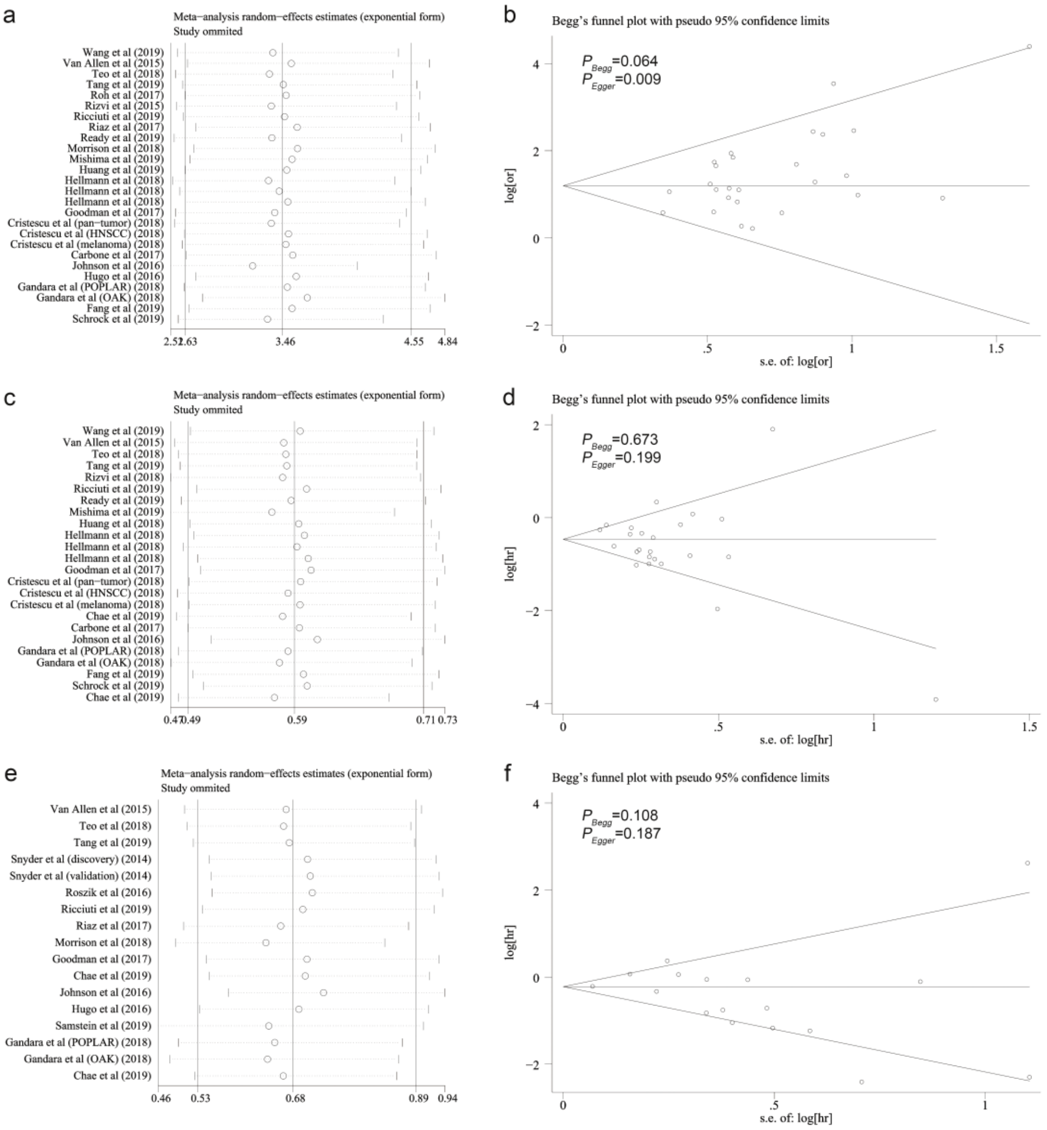

Figure S1. Sensitivity analyses and funnel plots in the meta-analysis. (a) sensitivity analysis and (b) funnel plot of pooled objective response rate. (c) sensitivity analysis and (d) funnel plot of pooled progression-free survival. (e) sensitivity analysis and (f) funnel plot of pooled overall survival.

Table S3. Additional results of subgroup analyses of the association between TMB and efficiency of immune checkpoint inhibitors

Categories	ORR				PFS				OS			
	number of articles (cohorts)	number of patients	Q test	Z test	number of articles (cohorts)	number of patients	Q test	Z test	number of articles (cohorts)	number of patients	Q test	Z test
**cancer type**												
non-small-cell lung cancer	8 (9)	919	.281	< .001	10 (11)	1164	.002	.001	5 (6)	869	.031	> .99
melanoma	8 (8)	448	.117	< .001	4 (4)	251	.012	.033	9 (10)	886	.001	.004
small cell lung cancer	2 (3)	194	.843	.002	2 (3)	194	.360	< .001	N/A			
urothelial carcinoma	N/A				N/A				2 (2)	425	.751	.657
gastroesophageal cancer	2 (2)	74	.746	.239	2 (2)	78	.054	.769	N/A			
others[a]	5 (6)	378	.399	< .001	5 (6)	386	.016	.009	4 (4)	756	.687	.001
**area**												
Western	15 (18)	1240	.120	< .001	13 (16)	1311	< .001	< .001	12 (12)	2400	< .001	.014
Asian	5 (5)	220	.619	.003	5 (5)	224	.028	.134	N/A			
others[a]	2 (3)	553	.559	< .001	2 (3)	538	.167	.006	3 (5)	516	.042	.214
**TMB sequencing method**												
WES	11 (14)	1038	.676	< .001	7 (10)	844	.463	< .001	4 (5)	279	.207	.003
targeted NGS	11 (12)	975	.036	< .001	13 (14)	1229	< .001	.010	11 (12)	2657	< .001	.064
**class of immune checkpoint inhibitors**												
anti-PD-(L)1	15 (19)	1531	.081	< .001	15 (18)	1445	< .001	< .001	9 (10)	814	.001	.153

anti-CTLA-4	2 (2)	126	> .99	.080		N/A			4 (5)	349	.163	4 (5)
anti-PD-(L)1 plus anti-CTLA-4	3 (3)	226	.597	< .001	3 (3)	226	.333	< .001		N/A		
others[a]	2 (2)	130	.499	.084	3 (3)	402	.082	.030	3 (3)	1872	.024	.554
**line of therapy**												
1	2 (2)	256	.286	< .001	2 (2)	207	.489	.002		N/A		
others[a]	20 (24)	1757	.149	< .001	18 (22)	1866	< .001	< .001	15 (17)	2936	< .001	.004

[a]others included subgroups with only one report and articles containing multiple subgroups which could not be further subdivided. Abbreviations: ORR: objective response rate/overall response rate. PFS: progression-free survival. OS: overall survival. TMB: tumor mutation burden. WES: whole exome sequencing. NGS: next generation sequencing. PD-(L)1: programmed cell death 1/programmed cell death ligand 1. CTLA-4: cytotoxic T-lymphocyte-associated protein 4. N/A: not applicable.

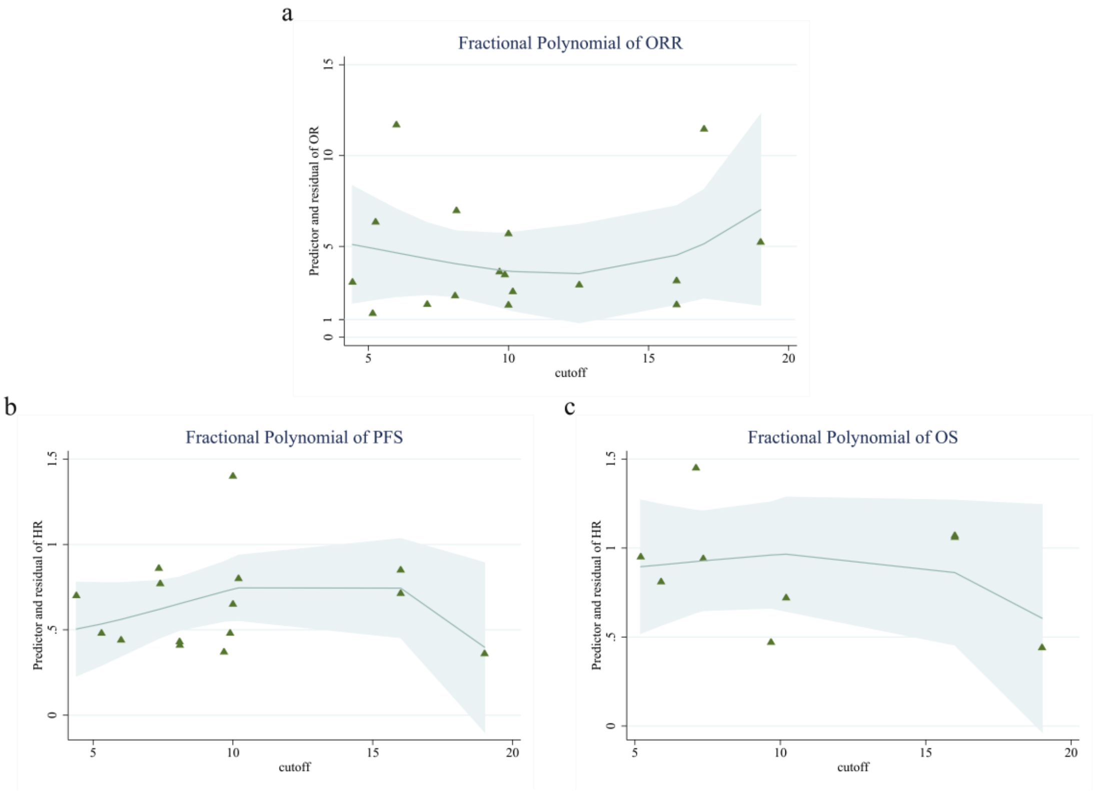

Figure S2. Fractional polynomial regression of TMB and clinical endpoints of ICIs. Fractional polynomial of (a) ORR, (b) PFS, (c) OS. Abbreviations: ORR: objective response rate/overall response rate. PFS: progression-free survival. OS: overall survival. OR: odds ratio. HR: hazard ratio. TMB: tumor mutation burden.

Table S4. Additional results of combined analysis of TMB and PD-L1 expression on objective response rate of immune checkpoint inhibitors

subgroup	TMB high vs TMB low			PD-L1 high vs PD-L1 low		
	number of articles (cohorts)	Z test	Q test	number of articles (cohorts)	Z test	Q test
PD-L1 high group	9 (11)	< .001	0.505	N/A		
PD-L1 low group	8 (9)	< .001	0.807			
TMB high group	N/A			9 (11)	0.002	0.386
TMB low group				9 (11)	< .001	0.975

Abbreviations: TMB: tumor mutation burden. PD-L1: programmed cell death ligand 1. N/A: not applicable.
